# Prognostic value and susceptibility of *BAX* rs4645878 polymorphism in cancer: A systematic review and meta-analysis

**DOI:** 10.1097/MD.0000000000011591

**Published:** 2018-07-20

**Authors:** Ye Feng, Xianglei Chen, Yi Zheng, Qiao Liu, Haiwen Chen, Yuanhua Cai, Lixia Cao, Xiaolin Lai, Lili Pan, Yang Li, Shao-Yuan Wang

**Affiliations:** a Fujian Institute of Hematology, Fujian Provincial Key Laboratory on Hematology, Fujian Medical University Union Hospital, Fuzhou City; b Union Clinical Medical Colleges, Fujian Medical University; c Department of hematology, Fujian Fuzhou Children's Hospital, Fuzhou, Fujian Province, PR China.

**Keywords:** *BAX*, cancer, gene polymorphism, meta-analysis, prognosis, susceptibility

## Abstract

**Background:**

*BCL-2 Associated X* (*BAX*) is an important modulator of apoptosis. The associations between *BAX* gene polymorphism and cancer susceptibility and prognosis in different ethnic groups and types of cancer have yielded controversial results. To reconcile the results, a systematic review followed by meta-analysis was performed to assess the associations.

**Methods:**

A systematic search of Medline database (PubMed), EMBASE, China Biology Medicine disc, China National Knowledge Infrastructure, Wanfang databases for publications on *BAX* polymorphisms, and susceptibility and prognosis was carried out until July 2017. Retrieved 14 articles met the inclusions. Summary odds ratios (ORs) and hazard ratios (HRs) with their 95% confidence intervals (CIs) were harnessed to determine the strength of correlation between *BAX* polymorphisms and cancer susceptibility and prognosis, which were combined using fixed- or random-effects models as appropriate.

**Results:**

A total of 12 trials involving 3321 cases and 3209 controls were included in our pooled analysis regarding the polymorphisms and the susceptibility of cancers. Overall, results of the present meta-analysis demonstrated that there was no significant association between *BAX* polymorphisms and susceptibility of cancers (OR = 1.052, 95% CI: 0.827–1.339, *P* = .679, A vs G). Even in a stratified analysis by ethnicity and the sources of control groups, the results were consistent. Four retrospective studies of 549 cases qualified for meta-analysis were identified to set forth the associations of the polymorphisms with cancer prognosis. Our results suggested that *BAX* gene polymorphisms were significantly associated with unfavorable prognosis (HR = 1.735, 95% CI: 1.368–2.202, *P* = .000, GG vs GA/AA).

**Conclusion:**

There is no significant association between *BAX* gene polymorphism and cancer susceptibility, but it probably contributes to increased adverse prognosis to cancer.

## Abstract 2

Supplemental Digital Content is available in the text

## 1 Introduction

The formation of cancer is the process of multiple stages and factors that interact with environment and gene. Apoptosis is the basic biological process, which removes excess or aberrant cells in multicellular organisms and plays a crucial role in individual growth, development, and other stages of life. Lack of apoptosis alters the inter-cell homeostasis that may lead to tumorigenesis and promote tumor progression. There are 3 pathways leading to apoptosis: endogenous mitochondrial pathway, apoptotic pathway mediated by death receptor, and apoptotic pathway mediated by B granzyme signaling pathway.^[1]^ In the endogenous pathway, mitochondrial outer membrane permeabilization leads to the release of cytochrome *c* into the cytoplasm, which is controlled mainly by proteins in the upstream of the Bcl-2 family.^[2]^ The Bcl-2 family is a group of highly conserved evolutionarily and apoptotic-related proteins. The Bcl-2 family mainly includes pro-apoptotic members, such as *BCL-2 Associated X* (*BAX*) and *BAK*, as well as antiapoptotic members, such as *BCL-2* and *BCL-XL*, whose relative expression determines the destiny of the cell. Under normal conditions, pro-apoptosis and antiapoptosis are in a dynamic equilibrium. When abnormal internal environment or external stimulation leads to an imbalance of pro-apoptosis and antiapoptosis, the body may not be compensated, which can lead to tumorigenesis.

The *BAX* gene is the apoptotic gene directly regulated by *P53*, located on the human chromosome 19q13.3, encoding 6 exons and 1 promoter.^[3]^ Bax promotes programmed cell death by the intrinsic pathway with a series of changes including conformational switching, trafficking, and aggregation status changes.^[4]^ The association between *BAX* gene polymorphisms and cancer is related not only to the formation of cancer, but also to the prognosis of cancer. Starczynski et al^[5]^ study showed that *BAX* single nucleotide polymorphism (SNP) led to decreased expression of Bax protein, and slow down the activation of P53 cell response, which results in a conformational change of Bax protein, the failure of the mitochondrial apoptotic pathway, leading to tumor development, progression, drug resistance, and poor prognosis. Mutations in the promoter region and coding region of the *BAX* gene have been recognized as altering protein expression and functions.^[6,7]^ Consequently, the analysis of the promoter region and coding region of the *BAX* gene plays a key role in understanding protein function.

Existing studies have debated whether the *BAX* gene polymorphism increased cancer susceptibility and suggested adverse outcomes. A Genome-Wide Association Studies of Zintzaras et al^[8]^ on *BAX* gene included 4 case–control studies, comprising of the Caucasian race only, showed that there was no remarkable correlation between *BAX* gene polymorphism and chronic lymphocytic leukemia (CLL).

Meta-analysis of Sahu et al^[9]^ embracing 7 studies, reported that the *BAX* polymorphism was not associated with tumor susceptibility. However, not only been the results unreliable because of out of the Hardy–Weinberg equilibrium (HWE) balance, but later studies^[10,11]^ have suggested that the polymorphism was associated with susceptibility to cancer. Besides, neither did they make the subgroup analysis, nor did they perform the prognosis analysis. Therefore, it is necessary to do the meta-analysis again. With the publication of new studies in recent years, an increasing number of articles have paid attention to the relationship between *BAX* gene polymorphism and prognosis of different tumors, but the results are conflicted. Starczynski et al,^[5]^ Peng et al,^[12]^ and Brito et al^[13]^ affirmed the relationship between the polymorphism and prognosis, but Skogsberg et al^[14]^ defeated the conclusions. Furthermore, we have never seen any meta-analysis to assess the polymorphism associated with prognosis so far. In this study, we used meta-analysis method to make a comprehensive quantitative analysis in order to further clarify the relationship between *BAX* gene polymorphism and tumor susceptibility and prognosis.

## 2 Methods

### 2.1 Search strategy

Eligible studies were identified from computer-aided literature searches in Medline database (PubMed), EMBASE, China Biology Medicine disc, China National Knowledge Infrastructure, Wanfang databases for publications with the following Keywords: “BCL2-associated X” OR “*BAX*”) AND (“polymorphism(s)” OR “variant” OR “SNP”) AND (“tumor” OR “cancer” OR “carcinoma” OR “neoplasm” OR “malignancy”) up to July 2017, which involving the *BAX rs4546878* polymorphism with cancer risk and prognosis, exclusive of dissertations. The search strategy was conducted by reviewing potential articles and tracing the reference document.

### 2.2 Publication selection and quality assessment

The methodology of each study was assessed independently by 2 authors according to the Newcastle–Ottawa scale^[15]^ for assessing the quality of studies in meta-analysis. Each study was scored according to 3 aspects: selection, comparability, and exposure (0–4 scores for Grade 1 quality studies, 5–9 scores for grade 2 quality studies). Higher score is on behalf of better quality. Two reviewers independently screened the literature according to the inclusion criteria to complete information extraction and quality evaluation. Opinions that contained differences took the form of discussion or consultation with third parties to make trade-offs. The meta-analyses are based on previous published studies, thus no ethical approval and patients consent are required.

### 2.3 Data extraction and statistical analysis

The information extracted from the studies was the first author, published year, race, country, tumor type, genotype detection method, sample size, control group origin, the number of case groups and control groups, hazard ratio (HR), 95% confidence interval (CI), etc.

The pooled ORs with 95% CIs were calculated for each gene model (allelic model A vs G, dominant model AA + GA vs GG, implicit model AA vs GA + GG, additive model AA vs GG) to measure the strength of correlation between *BAX* gene polymorphisms and cancer susceptibility. The distribution of genotypes in controls was calculated for departure from HWE. Subgroup analyses were harnessed according to ethnicity and population sources to identify the specific effects of heterogeneity. Publication bias was tested by visual inspection of symmetry funnel plots and formally assessed by Begg and Egger tests. Sensitivity analysis was also utilized to confirm the stability of our findings.

Prognosis was estimated with overall survival (OS). OS was a defined period of time when someone has survived since treatment. We assessed the effect of *BAX* polymorphisms on prognosis by HR. For each study, the HR and its 95% CI of the dominant gene model (AG/AA vs GG) were retrieved. If these parameters were not available in studies, we used the software Engauge Digitizer 4.1 (http://sourceforge.net/projects/digitizer) to extract the specific survival rates according to the Kaplan–Meier curves to reconstruct survival curve and then calculate the HR by the methods described by Tierney et al^[16]^ sharing. Owing to the limited number of studies included in the analysis, publication bias and subgroup analysis were unable to implement.

All statistical analyses were carried out using STATA 12.0 software (STATA, College Station, TX). A *P* < .05 (2-tailed) was accepted as statistically significant. Statistical heterogeneity among the studies was detected using the Q test and I^2^ test. I^2^ < 25 indicates low heterogeneity, 25% ≤ I^2^ ≤ 50% indicates moderate heterogeneity, and I^2^ > 50% indicates large heterogeneity. When I^2^ > 50% or *P* < .10 (2-sided), the random-effects model (the DerSimonian–Laird method) was utilized to pool the data.^[17,18]^ Otherwise, the fixed-effects model (the Mantel–Haenszel method) was used.^[19]^

## 3 Results

### 3.1 Articles identification and selection

**Figure 1:**
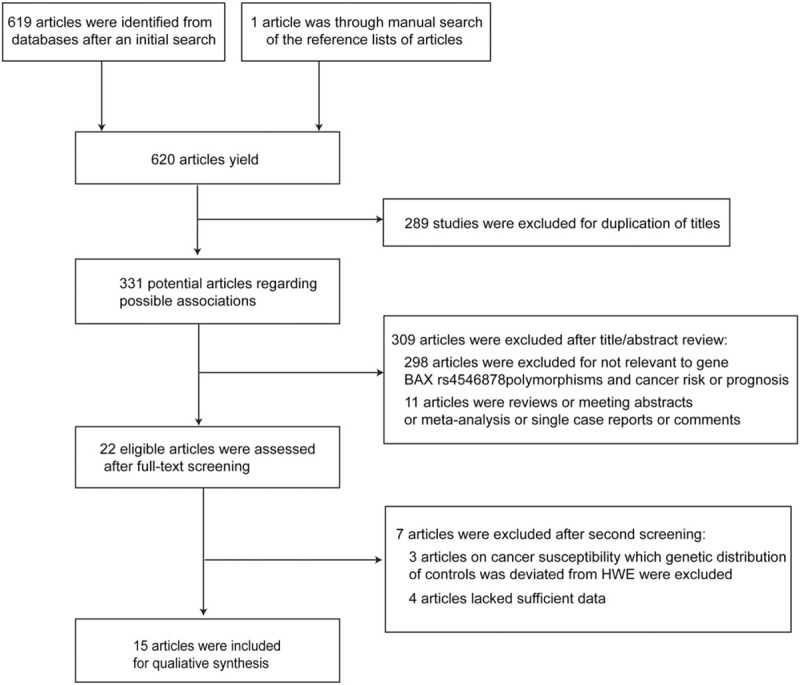
Flow chart of literature search and selection. *BAX* = *BCL-2 Associated X*, HWE = Hardy–Weinberg equilibrium.

The procedure of studies retrieval was shown in a flowchart (Fig. 1). Initially, 620 papers related to the keywords were identified. Three hundred thirty-one articles remained after duplicates removed. Then, 22 articles were assessed for eligibility through full-text reading. Owing to genetic distribution of controls deviating from HWE and insufficient data, 7 articles were excluded. In the end, after serious of filters were applied, 14 eligible studies were recruited, including 12 studies^[5,10,11,14,20–27]^ for susceptibility (3321 cases in case group, 3209 cases in control group), whose control groups were in line with HWE balance; 5^[5,12–14,28]^ for prognosis, among which 1^[28]^ was excluded because of greater heterogeneity, remaining 4 cases (549 cases). The characteristics and genotype distribution of the included analyses are presented in Tables 1, 2, and S1.

**Table 1:**
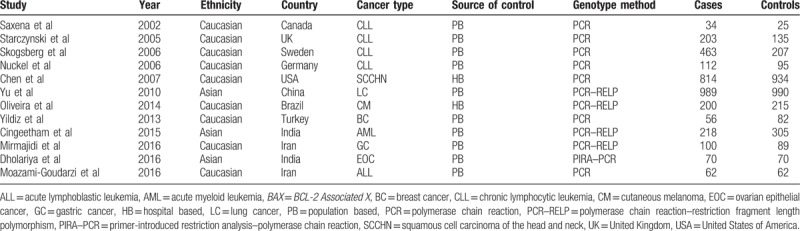
Characteristics of the individual studies included between the *BAX* rs4645878 polymorphism and the risk of cancer.

**Table 2:**
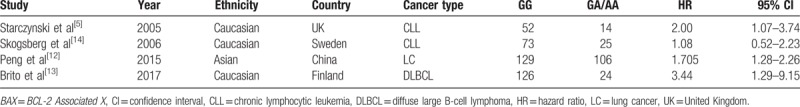
Characteristics of the individual studies included between the *BAX rs4645878* polymorphism and the prognosis of cancer.

### 3.2 Meta-analysis of relationship between *BAX* polymorphisms and susceptibility in cancers

**Figure 2:**
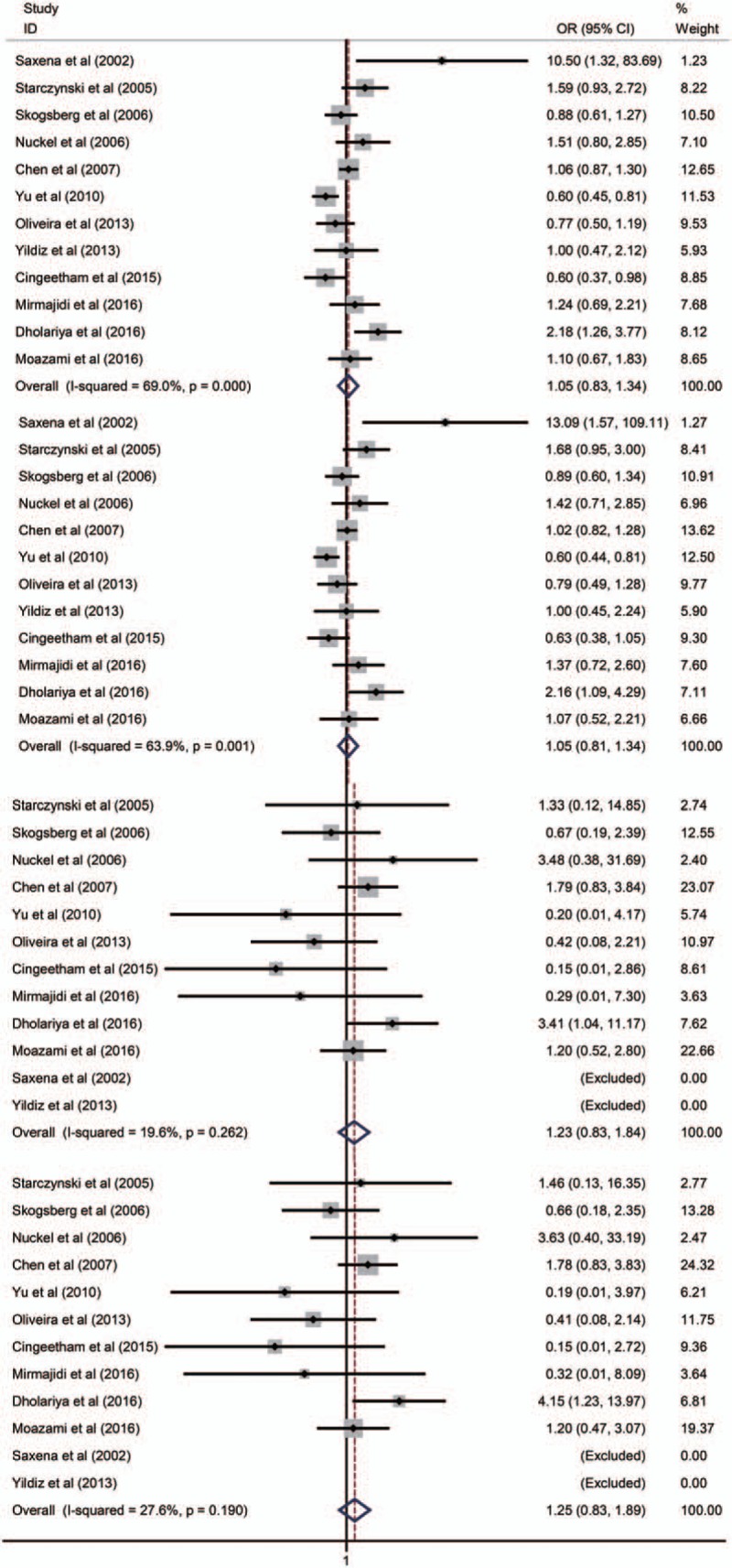
Forest plots of OR for cancer risk with *BAX rs4645878* polymorphism. A vs G, AA + GA vs GG were estimated with random-effect model. AA vs GA + GG, AA vs GG were estimated with fixed-effect model. *BAX* = *BCL-2 Associated X*, CI = confidence interval, OR = odds ratio.

Twelve case–control studies with 3321 patients and 3209 controls were included in the present meta-analysis of OR. Nine case–control studies were from Caucasians, 3 studies were from Asians. Overall, there was null collection of *BAX* polymorphisms in the allele frequencies or other genotype models with overall cancer risk (Fig. 2). Either in a subgroup analysis by ethnicity or by population source, no significant cancer risk was identified. Other comparison results are listed (Tables 3, S2, S3, and S4). For limited data, further subgroup analyses were not carried out.

**Table 3:**
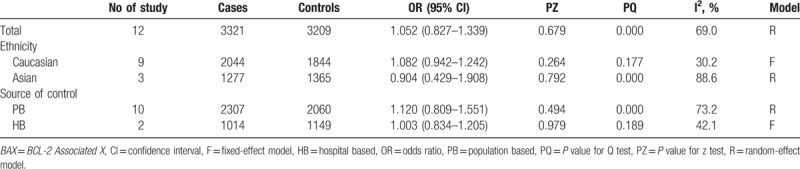
Meta-analysis *BAX* rs4645878 polymorphism and cancer risk (G vs A).

### 3.3 Meta-analysis of relationship between *BAX* polymorphisms and prognosis in cancers

**Figure 3:**
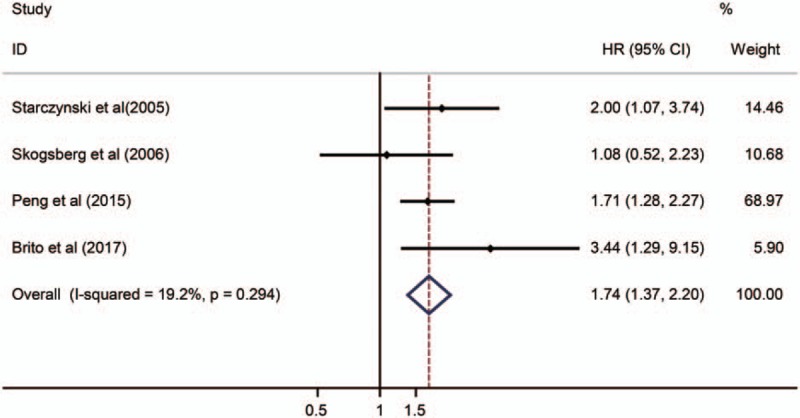
Forest plots of overall meta-analysis with fixed-effect estimates presenting HRs of cancer OS for *BAX rs4645878* (AA + GA vs GG). *BAX* = *BCL-2 Associated X*, CI = confidence interval, HR = hazard ratio, OS = overall survival.

Four studies with 549 patients were enrolled in the meta-analysis of OS. Three studies reported the association between *BAX* SNPs and OS but one without. Overall, *BAX* SNPs is correlated with poor OS (HR = 1.735, 95% CI: 1.368–2.202, *P* = .000, AA + GA vs GG) in cancer, which indicates that someone who carries at least one variant gene has a negative impact on the survival (Fig. 3).

### 3.4 Tests for publication bias, sensitivity analyses, and heterogeneity

Publication bias was assessed by visual inspection of funnel plots in Begg test and then estimated with bias *P* value from Egger and Begg tests in overall meta-analysis of OR. No significant publication bias was detected by Begg funnel plots (no apparent asymmetry was found) and estimation of *P* values (Fig. S1). We carried out a sensitivity analysis to evaluate the influence of any study on the pooled OR by omitting an individual study in sequence. The exclusion of anyone did not alter the corresponding pooled OR (Fig. S2). Heterogeneity across the studies was significant in analyzing association between the SNPs and the susceptibility (Tables 3, S2, S3, and S4). Thus, we evaluated the sources of heterogeneity by subgroup analysis. The results demonstrated that Asian populations may contribute to the major sources of heterogeneity.

**Figure 4:**
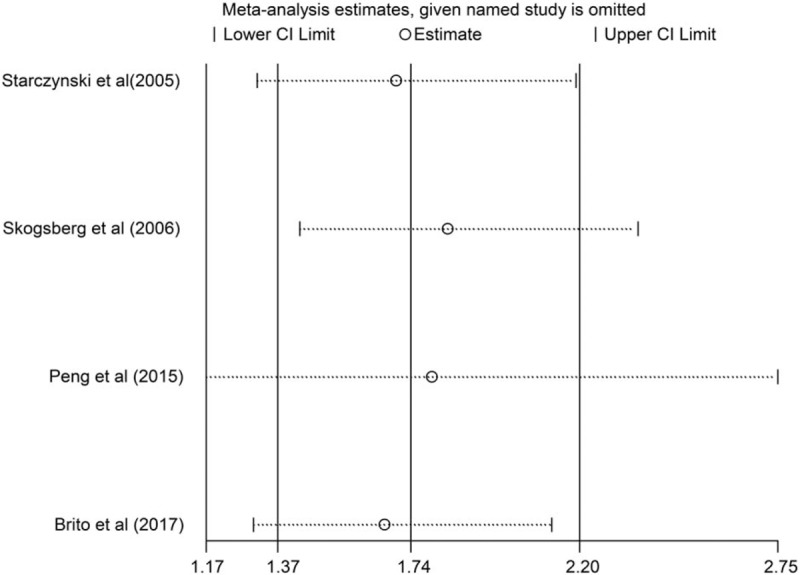
Sensitivity analysis of the influence between the *BAX rs4645878* polymorphism and the prognosis with fixed-effect estimates (AA + GA vs GG). *BAX* = *BCL-2 Associated X*.

An independent study involved in the present pooled-analysis was elided each time to assess the influence on the pooled HR. The results highlighted that our findings were relatively robust and reliable (Fig. 4). The heterogeneity between studies in analyses of prognosis was acceptable (GG vs GA/AA: χ^2^ = 3.71, *P* = .294, I^2^ = 19.2) (Fig. 3).

## 4 Discussion

*BAX*, as a member of pro-apoptotic gene in BCL-2 family, mainly is a pro-apoptotic role in the mitochondrial pathway. *BAX* promotes mitochondrial permeability transition pore (MPTP) opening. MPTP can be used as stimulation receptor of mitochondrial and receive some receptor-related signals. It includes the adenine nucleotides locating in the intermembrane space and adventitia-dependent ion channels.^[26]^ While *BCL-2* and *BCL-XL* inhibit PTP opening, then block the release of cytochrome *c* from mitochondria and hold back apoptosis.^[29,30]^ The imbalance of antiapoptosis and pro-apoptosis gives rise to tumorigenesis. The expression of Bax protein plays an important role in the release of apoptosis-related factors. Saxena et al^[20]^ demonstrated that *BAX G (-248) A* was related to protein expression. Yet the *BAX G (-248) A* polymorphism is controversial in protein expression. Compared with the A allele, Starczynski et al^[5]^ reported that G alleles were associated with higher mRNA and protein levels. Yu et al^[23]^ thought it had lower transcriptional activity. Skogsberg et al^[14]^ showed that there was no association between them. Low expression of Bax protein suggests poor prognosis.^[31]^ Whether its polymorphism could increase the susceptibility and work on the prognosis of the tumor. Different studies had supplied diverse conclusions. For this purpose, we have done this meta-analysis.

The relationship between *BAX* gene polymorphism and cancer prognosis has attracted more and more scholars’ attention. Some scholars have found that mutation of the gene locus led to Bax protein decreased expression, which is closely related to drug resistance. Such is why *BAX* gene polymorphism has a poor prognosis.^[31]^ While Skogsberg et al^[14]^ found that the *BAX* polymorphism is not associated with the prognosis of cancer patients. CLL is a kind of disease, which is commonly regarded as micro-RNA mediated over-expression of Bcl-2 protein caused by deletion of *BCL-2* gene expression suppression.^[32,33]^ Recently, a highly potent and selective oral Bcl-2 antagonist, venetoclax, has gotten the green light in CLL patients. The treatment of this disease by Bcl-2 antagonists is inspired by the important role of *BCL-2* in B-cell lymphoma pathophysiology.^[34]^ SMBA is Bax protein agonist that inhibits tumor growth by specifically acting on the S184 phosphorylated site of Bax.^[35]^ As a new anticancer drug, Bax protein agonist provides a new strategy for the treatment of malignant tumors expressed by *BAX* since its naissance.^[4,35]^ The drug has been validated in animal living and has not been tested in humans. In view of the important role of *BAX* gene in the formation and development of malignant tumor, Bax protein agonist is a very promising drug, warding off drug resistance, selectively inducing cancer cell apoptosis, and low toxicity in normal cells.^[4,35]^ Our study suggests that the *BAX* rs4645878 gene polymorphism is associated with adverse outcomes, so patients with this gene polymorphism may be an appropriate group for these drugs.

The association between *BAX* gene polymorphisms and cancer susceptibility has been reported. Most scholars have shown that *BAX* polymorphism is not related to cancer susceptibility, and a small amount of *BAX* gene polymorphism is associated with cancer susceptibility. Although the *BAX* gene polymorphism in our study on whether increasing cancer susceptibility was not statistically significant, and the results are consistent even with subgroup analyses based on race and control group sources, we still cannot ignore the interaction between it and other genes on cancer susceptibility, such as *TP53*, *BCL2*, and so on.

Sahu and Choudhuri^[9]^ meta-analysis results in 2013 showed that *BAX* gene polymorphism and susceptibility confirmed to be not associated with each other, which is consistent with our study. But in comparison with the previous meta-analyses, some advantages of the current study should be adequately addressed. Our study updated the data on *BAX* polymorphism and the risk of cancer. The types of cancer involved were more extensive than before. Besides, our results corroborating *BAX* polymorphism effected on cancer prognosis for the first time. Moreover, this study included literature conducted the strict quality evaluation. Methodological issues have also been well explored (e.g., publication bias, sensitivity analysis, heterogeneity analysis). In addition, the controls in the study were in line with the HWE balance. Last but not least, it was carried out at the level of genotype and was analyzed by ethnic and population sources subgroup. Hospital-based case–control studies were prone to selection bias and poor representation, which may be only on behalf of a relatively small population. Groups who come to the hospital may have more disease exposure factors or elements, which affect the polymorphism of the gene than normal populations. Therefore, a subgroup analysis was conducted according to the population source of each study's control groups and found no effect on our results. These measures make the research more powerful and more specific.

Although considerable efforts were made to detect the possible association between *BAX* SNPs and cancer susceptibility and prognosis, caution must be dealt with in the interpretation of these findings because of the large heterogeneity or small sample size design in our study. In subgroup analyses about cancer susceptibility stratified by racial descent, respectively, this heterogeneity was reduced significantly or removed in some subgroups, inferring the relatively large heterogeneity mainly stemmed from differences of ethnicity. Meanwhile, the large heterogeneity might also have roots in innate deficiencies, such as small sample size design. Besides, only published studies were enrolled in the present study and positive or significant studies may stand a good chance of publishing, thus publication bias may inevitably exist. What's more, in some subgroup, there were only 2 case–control studies conducted in Africans and 1 in Asians, which may be a fluke or restrict the statistical power to detect a real influence. Finally, due to the lack of sufficient background data, our findings were based on unadjusted ORs and HRs with their 95% CIs, and we cannot correct the confounding effects of certain confounding factors, such as gender, ages, specified type of cancer, and so on. Nevertheless, for practical reasons, larger scale of studies assessing gene–gene, gene–environment interaction and incorporating with functional assessments are warranted to confirm or refute these findings.

## 5 Conclusion

In summary, despite its limitations, this meta-analysis suggests *BAX* SNPs and susceptibility lack obvious connection while it leads to worse OS in cancer populations. In the future, further extensive studies with larger sample sizes and wider range of tumor types should be performed to enrich the evidence of the association of *BAX* gene polymorphisms on cancer susceptibility and prognosis.

## Author contributions

**Conceptualization:** Xianglei Chen, Yuanhua Cai.

**Data curation:** Ye Feng, Yuanhua Cai, Lixia Cao.

**Formal analysis:** Ye Feng, Yi Zheng, Xiaolin Lai.

**Funding acquisition:** Qiao Liu, Shaoyuan Wang.

**Investigation:** Qiao Liu, Haiwen Chen.

**Methodology:** Ye Feng, Xianglei Chen, Lixia Cao.

**Project administration:** Qiao Liu.

**Resources:** Qiao Liu, Haiwen Chen.

**Software:** Ye Feng, Yi Zheng, Xiaolin Lai.

**Supervision:** Yi Zheng, Haiwen Chen, Yuanhua Cai, Lixia Cao, Lili Pan, Yang Li.

**Validation:** Yi Zheng, Haiwen Chen, Lili Pan.

**Visualization:** Yi Zheng, Yang Li.

**Writing – original draft:** Ye Feng, Lixia Cao, Xiaolin Lai.

**Writing – review and editing:** Ye Feng, Xianglei Chen, Lili Pan, Yang Li.

## Supplementary Material

Supplemental Digital Content
