# Supplementary material for: Prognostic value and susceptibility of BAX rs4645878 polymorphism in cancer: A systematic review and meta-analysis
Source: Medicine (Baltimore). 2018 Jul 20;97(29):e11591. doi: 10.1097/MD.0000000000011591 (PMC6086507; doi:10.1097/MD.0000000000011591)
Supplement: Supplemental Digital Content [file medi-97-e11591-s001.docx]

Figure S1 Publication bias test for the correlations of *BAX rs4645878* polymorphism with cancer risk（A vs G）.


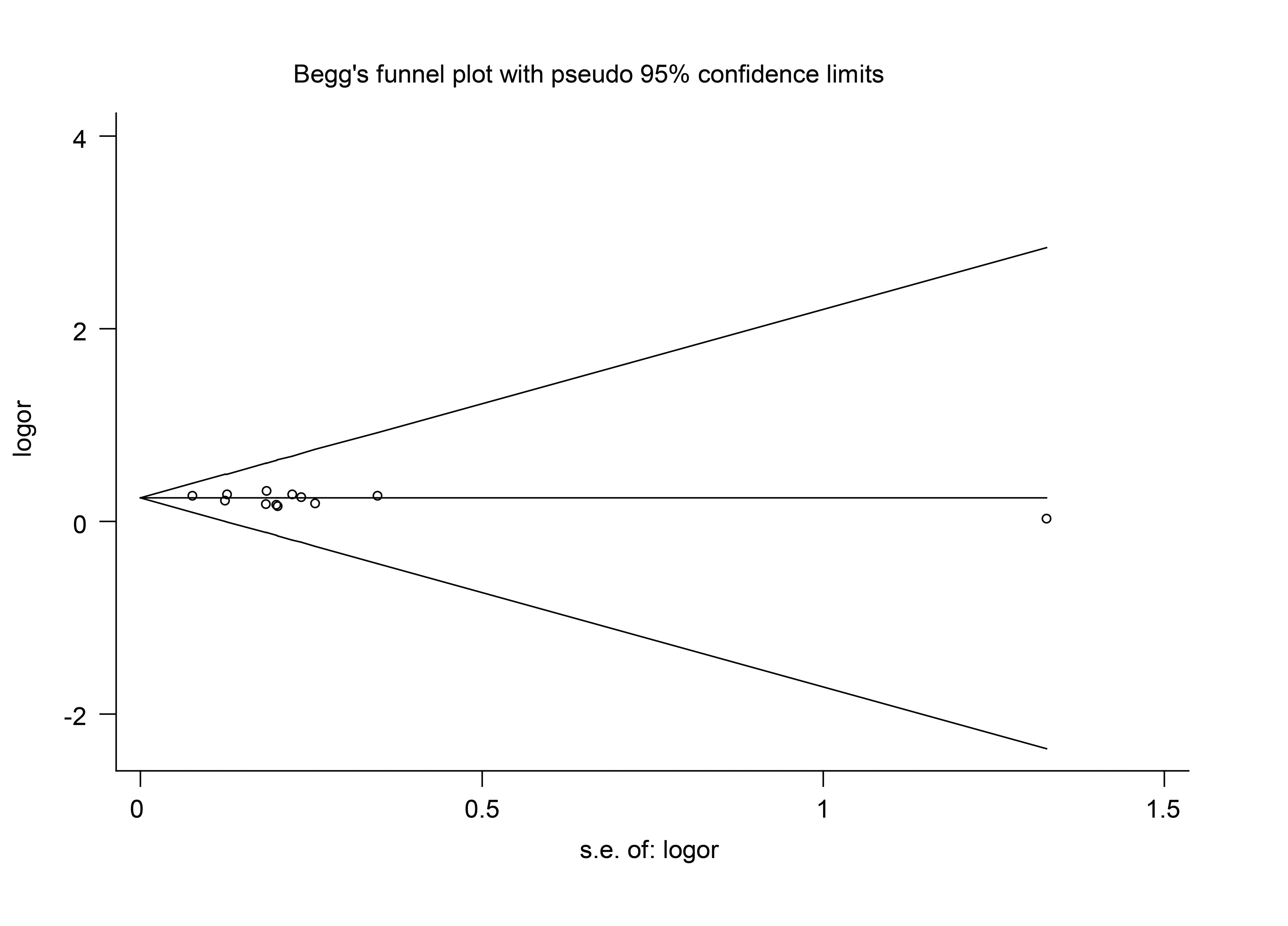


Abbreviation：*BAX=BCL-2 Associated X*

Figure S2 Sensitivity analysis of the influence between the *BAX rs4645878* polymorphism and the risk of cancer with a random-effects estimates（A vs G）


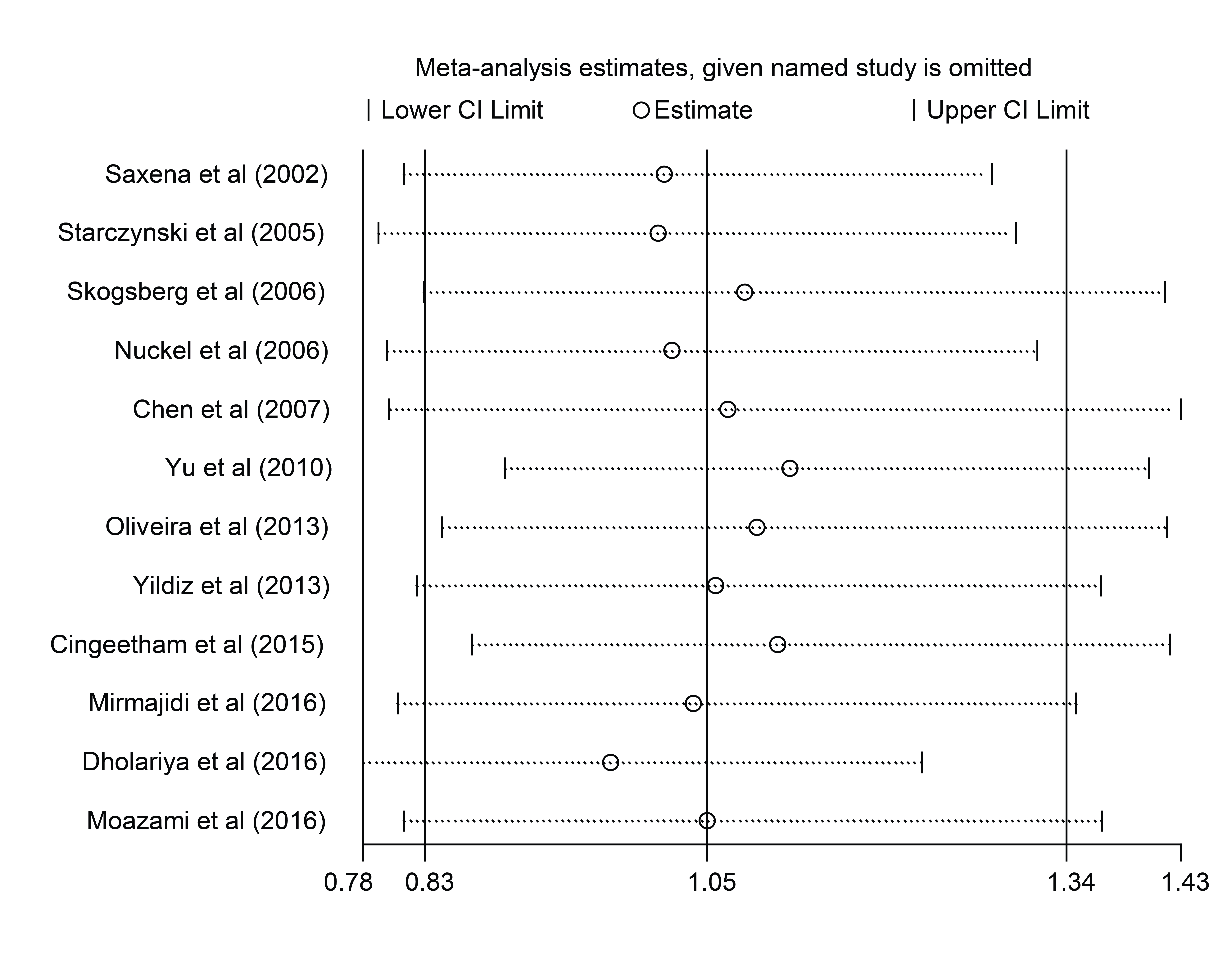


Abbreviation：*BAX=BCL-2 Associated X*

**Table S1 Distribution of *BAX*** **rs4645878 polymorphisms genotype**

| **stydy** | **year** | **cases** | | | **controls** | | | **cases** | | **controls** | | **HWE** |
| --- | --- | --- | --- | --- | --- | --- | --- | --- | --- | --- | --- | --- |
|  |  | **GG** | **GA** | **AA** | **GG** | **GA** | **AA** | **G** | **A** | **G** | **A** |  |
| Saxena et al [17] | **2002** | **22** | **12** | **0** | **24** | **1** | **0** | **56** | **12** | **49** | **1** | **YES** |
| Starczynski et al [5] | **2005** | **157** | **44** | **2** | **115** | **19** | **1** | **358** | **48** | **249** | **21** | **YES** |
| Skogsberg et al [14] | **2006** | **373** | **84** | **6** | **163** | **40** | **4** | **830** | **96** | **366** | **48** | **YES** |
| Nuckel et al [18] | **2006** | **87** | **21** | **4** | **79** | **15** | **1** | **195** | **29** | **173** | **17** | **YES** |
| Chen et al [19] | **2007** | **627** | **170** | **17** | **723** | **200** | **11** | **1424** | **204** | **1646** | **222** | **YES** |
| Yu et al [20] | **2010** | **913** | **76** | **0** | **869** | **119** | **2** | **1902** | **76** | **1857** | **123** | **YES** |
| Oliveira et al [22] | **2013** | **163** | **35** | **2** | **167** | **43** | **5** | **361** | **39** | **377** | **53** | **YES** |
| Yildiz et al [21] | **2013** | **43** | **13** | **0** | **63** | **19** | **0** | **99** | **13** | **145** | **19** | **YES** |
| Cingeetham et al [10] | **2015** | **193** | **25** | **0** | **253** | **48** | **4** | **411** | **25** | **554** | **56** | **YES** |
| Mirmajidi et al [23] | **2016** | **69** | **31** | **0** | **67** | **21** | **1** | **169** | **31** | **155** | **23** | **YES** |
| Dholariya et al [11] | **2016** | **34** | **24** | **12** | **47** | **19** | **4** | **92** | **48** | **113** | **27** | **YES** |
| Moazami-Goudarzi et al [24] | **2016** | **23** | **24** | **15** | **24** | **25** | **13** | **70** | **54** | **73** | **51** | **YES** |

Abbreviation：*BAX=BCL-2 Associated X*; HWE= Hardy- Weinberg equilibrium.

Table S2 Meta-analysis *BAX* rs4645878 polymorphism and cancer risk（GG vs AA）

|  | No.of study | cases | controls | OR(95%CI) | PZ | PQ | I²² | model |
| --- | --- | --- | --- | --- | --- | --- | --- | --- |
| total | 10 | 1954 | 1737 | 1.252(0.832-1.885) | 0.282 | 0.190 | 27.6% | F |
| ethnicity |  |  |  |  |  |  |  |  |
| Caucasian | 7 | 1954 | 1737 | 1.216(0.761-1.943) | 0.413 | 0.528 | 0.0% | F |
| Asian | 3 | 1277 | 1365 | 0.654(0.051-8.411) | 0.745 | 0.029 | 71.7% | R |
| Source of control |  |  |  |  |  |  |  |  |
| PB | 8 | 2217 | 1953 | 1.205(0.719-2.021) | 0.479 | 0.195 | 29.2% | F |
| HB | 2 | 1014 | 1149 | 1.033(0.256-4.162) | 0.963 | 0.113 | 60.1% | R |

Abbreviation：*BAX=BCL-2 Associated X*; CI= confidence interval; PB= population based; HB=hospital based;

NA: not available; OR=odds ratio; PZ= P value for z test; PQ= P value for Q test; R= random effect model;

F= fixed effect model.

Table S3 Meta-analysis *BAX* rs4645878 polymorphism and cancer risk（GA+AA vs GG）

|  | No.of study | cases | controls | OR(95%CI) | PZ | PQ | I²² | model |
| --- | --- | --- | --- | --- | --- | --- | --- | --- |
| total | 12 | 3321 | 3209 | 1.046(0.815-1.342) | 0.726 | 0.001 | 63.9% | R |
| ethnicity |  |  |  |  |  |  |  |  |
| Caucasian | 9 | 2044 | 1844 | 1.078(0.925-1.258) | 0.337 | 0.178 | 30.1% | F |
| Asian | 3 | 1277 | 1365 | 0.882(0.449-1.733) | 0.001 | 0.003 | 82.8% | R |
| Source of control |  |  |  |  |  |  |  |  |
| PB | 10 | 2307 | 2060 | 1.127(0.801-1.585) | 0.491 | 0.001 | 69.4% | R |
| HB | 2 | 1014 | 1149 | 0.976(0.796-1.195) | 0.812 | 0.340 | 0.0% | F |

Abbreviation：*BAX=BCL-2 Associated X*; CI= confidence interval; PB= population based; HB=hospital based;

NA: not available; OR=odds ratio; PZ= P value for z test; PQ= P value for Q test; R= random effect model;

F= fixed effect model.

Table S4 Meta-analysis *BAX* rs4645878 polymorphism and cancer risk（AA vs GA+GG）

|  | No.of study | cases | controls | OR(95%CI) | PZ | PQ | I²² | model |
| --- | --- | --- | --- | --- | --- | --- | --- | --- |
| total | 10 | 3231 | 3102 | 1.231(0.825-1.837) | 0.308 | 0.262 | 19.6% | F |
| ethnicity |  |  |  |  |  |  |  |  |
| Caucasian | 7 | 1954 | 1737 | 1.213(0.770-1.912) | 0.406 | 0.540 | 0.0% | F |
| Asian | 3 | 1277 | 1365 | 0.655(0.062-6.875) | 0.725 | 0.048 | 67.0% | R |
| Source of control |  |  |  |  |  |  |  |  |
| PB | 8 | 2217 | 1953 | 1.170(0.711-1.927) | 0.537 | 0.275 | 19.5% | F |
| HB | 2 | 1014 | 1149 | 1.057(0.271-4.122) | 0.936 | 0.121 | 58.5% | R |

Abbreviation：*BAX=BCL-2 Associated X*; OR=odds ratio; CI= confidence interval; PB= population based;

HB=hospital based; NA: not available; PZ= P value for z test; PQ= P value for Q test; R= random

effect model; F= fixed effect model.
